# Metabolite-Sensing G Protein Coupled Receptor TGR5 Protects Host From Viral Infection Through Amplifying Type I Interferon Responses

**DOI:** 10.3389/fimmu.2018.02289

**Published:** 2018-10-02

**Authors:** Qingqing Xiong, Hongjun Huang, Ning Wang, Ruoyu Chen, Naiyang Chen, Honghui Han, Qin Wang, Stefan Siwko, Mingyao Liu, Min Qian, Bing Du

**Affiliations:** ^1^Shanghai Key Laboratory of Regulatory Biology, Institute of Biomedical Sciences and School of Life Sciences, East China Normal University, Shanghai, China; ^2^Department of Nephrology and Rheumatology, Shanghai Fengxian Central Hospital, Shanghai, China; ^3^Institute of Biosciences and Technology, Department of Molecular and Cellular Medicine, Texas A&M University Health Science Center, Houston, TX, United States

**Keywords:** TGR5, ISG, metabolite-sensing GPCRs, IFN-I, viral infection

## Abstract

The metabolite-sensing G protein–coupled receptors (GPCRs) bind to various metabolites and transmit signals that are important for proper immune and metabolic functions. However, the roles of metabolite-sensing GPCRs in viral infection are not well characterized. Here, we identified metabolite-sensing GPCR TGR5 as an interferon (IFN)-stimulated gene (ISG) which had increased expression following viral infection or IFN-β stimulation in a STAT1-dependent manner. Most importantly, overexpression of TGR5 or treatment with the modified bile acid INT-777 broadly protected host cells from vesicular stomatitis virus (VSV), newcastle disease virus (NDV) and herpes simplex virus type 1 (HSV-1) infection. Furthermore, VSV and HSV-1 replication was increased significantly in *Tgr5*-deficient macrophages and the VSV distribution in liver, spleen and lungs was increased in *Tgr5*-deficient mice during VSV infection. Accordingly, *Tgr5*-deficient mice were much more susceptible to VSV infection than wild-type mice. Mechanistically, TGR5 facilitates type I interferon (IFN-I) production through the AKT/IRF3-signaling pathway, which is crucial in promoting antiviral innate immunity. Taken together, our data reveal a positive feedback loop regulating IRF3 signaling and suggest a potential therapeutic role for metabolite-sensing GPCRs in controlling viral diseases.

## Introduction

Diet and its metabolites, as well as bacterial metabolites, are increasingly recognized for their important roles in the immune system. Furthermore, microbial associated metabolites are also involved in protection from influenza through augmentation of type I interferon (IFN-I) signaling (1). Since viral replication and spreading depends on host cell metabolic and biosynthetic machinery, viral infection will modify the cellular metabolism and associated signaling pathways. Therefore an analysis of the metabolites and their receptors modulated by immune function may be crucial for elucidating new anti-viral host defenses (2).

With nearly 1,000 members, G protein-coupled receptors (GPCRs) constitute the most diverse class of sensory receptors, able to recognize neurotransmitters, hormones, ions, amino acids, and other extracellular stimuli (3, 4). More and more GPCRs including free fatty acid receptors (5), purinergic receptors (6), adenosine receptors (7), dopamine receptors (8), and R-spondins receptors (9) have been found to regulate immune responses. Not surprisingly, these receptors carry out a multitude of tasks in viral infection. For example, CXC-chemokine receptor-4 (CXCR4) and CC-chemokine receptor-5 (CCR5) are cell fusion co-receptors for HIV infection (10). Meanwhile, our previous studies have demonstrated that P2Y_6_ (11), P2Y_13_ (12), GPR146 (13), LGR4 (14), and GPR54 (15) are all related to viral infection in distinctive manners. Thus, identification of additional GPCRs in viral infection and related immune responses may be clinically important in the prevention and control of viral infectious diseases.

As a member of metabolite-sensing GPCRs, the G protein-coupled bile acids receptor, GPBAR1 (TGR5), was discovered in 2002 (16). At the time, TGR5 was widely studied as a metabolic regulator involved in bile acids synthesis, glucose metabolism and energy homeostasis (17). Different from other nuclear receptors for bile acids, TGR5 is abundantly expressed in monocytes/macrophages and responsive to bile acids as a cell-surface receptor (18). More recently, TGR5 has been extended to function in cancer, liver regeneration and inflammatory responses (19). However, as the bile acid membrane receptor, the role and mechanism of TGR5 in viral infection remains unidentified although bile acids have been found to restrict rotavirus and hepatitis B virus infection through the nuclear farnesoid X receptor (FXR) (20, 21). In the present study, we demonstrated that metabolite-sensing TGR5 could be regarded as an ISG in viral infection which inhibited viral propagation by promoting IFN-I production via AKT-mediated IRF3 activation. Thus, our findings reveal a new positive feedback regulatory mechanism for IFN-I signaling in antiviral innate immune responses and suggest a potential therapeutic role for TGR5 in treating and preventing viral infection.

## Materials and methods

### Mice

*Tgr5*-knockout mice (*Tgr5*^−/−^) on a C57BL/6 background were kindly provided by Professor Jian Luo (East China Normal University). Sequences for primers used for confirming the mutated mice are 5′-GATAATGTGCTGTCCCCACC-3′ (forward) and 5′-AGCTGACCCAGGTGAGGAAC-3′ (reverse). All mice were bred in specific pathogen-free conditions and all animal experiments were approved by the East China Normal University Center for Animal Research.

### Chemicals and reagents

DMEM medium, RPMI-1640, penicillin-streptomycin and Lipofectamine 2000 were acquired from Invitrogen Life Technologies. Fetal bovine serum (FBS) was purchased from HyClone. Polyinosine-polycytidylic acid [Poly (I:C)] was obtained from Invivogen. Lipopolysaccharide (LPS) and M2 beads were purchased from Sigma. Recombinant mouse IFN-β was obtained from Sino Biological. TRIzol reagent and PrimeScript RT Master Mix were purchased from Takara. SYBR Green PCR Master Mix was purchased from Yeasen. Dual-Luciferase Reporter assay reagent was purchased from Promega. Antibodies specific to TBK1, phosphorylated TBK1, IRF3, phosphorylated IRF3, ERK, phosphorylated ERK, P38, phosphorylated P38, JNK, phosphorylated JNK, AKT, and phosphorylated AKT were obtained from Cell Signaling Technology. Polyclonal anti-GAPDH, anti-HA, anti-Flag antibody were obtained from Biogot technology. HA beads were obtained from Abmart. INT-777 (a novel potent and selective TGR5 agonist) was purchased from MedChem Express.

### Cell collection and culture

HEK-293T, Vero, and RAW264.7 cells were purchased from the American Type Culture Collection and cultured in complete DMEM containing 10% FBS and 1% penicillin-streptomycin. Peritoneal macrophages (PEMs) and bone marrow-derived macrophages (BMMs) were isolated and cultured as previously described (22).

### Virus propagation and plaque assays

Herpes simplex virus type 1 (HSV-1), Indiana serotype of VSV, NDV-GFP virus, and VSV-GFP virus were propagated in Vero cells and the titers were determined by standard plaque assays. Briefly, collected viruses were serially diluted and infected in Vero cells for 1 h. Then the infected cells were covered with growth medium containing 1.5% (mas/vol) low-melting point agarose. Plaques were counted after 16 h or 48 h for VSV/NDV and HSV-1, respectively.

### Plasmids and transfection

GFP-TGR5 plasmid was obtained from GeneCopoeia. IFN-β-luciferase reporter, Renilla, Flag-RIG-I (N), Flag-MAVS, Flag-STING, Flag-IRF3, HA-AKT, and Flag-TBK1 were kindly provided by Professor Ping Wang (Tongji University). Flag-IRF3-5D mutant plasmid was a gift from Professor Dong Xie (Institute for Nutritional Sciences, Shanghai Institutes for Biological Sciences, Chinese Academy of Sciences). Transfections were performed using calcium phosphate-DNA co-precipitation method (HEK-293T cells) or Lipofectamine 2000 (RAW264.7 cells) and cells transfected with the same amount of empty vector (Emv) were used as control.

### RNA interference

PEMs were seeded into 12-well plates at 1 × 10^6^ cells per well and transfected with 50 nM *Stat1* small interfering RNA (*Stat1* siRNA) duplexes (with the following siRNA sequences: 5′-GGAAAAGCAAGCGUAAUCUTT-3′) using Lipofectamine 2000 according to the manufacturer's instructions. PEMs transfected with the same amount of universal non-targeting siRNA were used as negative control (si NC).

### Real-time quantitative PCR

Total RNA was extracted with TRIzol reagent (Takara) and reversed-transcribed using the PrimeScript RT Master Mix Kit (Takara). The reverse transcription products were used as templates and subjected to quantitative PCR (Q-PCR). The primer sequences for Q-PCR analysis are listed in Supplementary Material (Table S1).

### Western blots and immunoprecipitation

Cells were lysed using RIPA buffer containing protease inhibitors and cleared by centrifugation. Protein concentrations of the extracts were measured by BCA assay (Pierce) and equalized with the extraction reagent. Samples were loaded and heated for 15 min at 100°C, separated by SDS–PAGE, transferred onto nitrocellulose membranes, blocked with 5% bovine serum albumin, followed by incubation with primary antibodies overnight and then incubated with the anti-mouse or anti-rabbit fluorescent secondary antibodies. The immunoreactive bands were visualized by the Odyssey system (LI-COR Biosciences). For coimmunoprecipitation assays, cell extracts were incubated with M2 beads for 3 h at 4°C. The immunoprecipitates were washed three times with the lysis buffer and subjected to immunoblot analysis.

### Luciferase reporter assays

HEK-293T cells were transfected with IFN-β luciferase reporter plasmids together with Renilla plasmids and other described plasmids for 28 h. Then the cells were lysed and assayed for luciferase activity using the Dual-Luciferase Assay Kit (Promega), according to the manufacturer's protocol. Reporter luciferase activity was determined by normalizing Firefly luciferase activity to Renilla luciferase activity.

### Lung histology

Lungs from control or virus-infected mice were dissected, fixed in 4% paraformaldehyde, embedded into paraffin, cut into sections, stained with haematoxylin & eosin solution, and examined by light microscopy.

### Flow cytometry

PEMs were infected with VSV-GFP (0.01 MOI) for 12 h and resuspended in 200 μl of phosphate buffered saline (PBS). Then VSV-GFP was measured by FACSCalibur flow cytometer (BD biosciences) and the data were analyzed with FlowJo software.

### Statistical analysis

Statistical analyses were performed by Student's *t*-test using Prism 5.0 (GraphPad Software). The values are shown as the mean ± SD. Kaplan–Meier curves present mouse survival rates and the values are expressed as the mean ± SEM of n animals. Statistical values where *P* < 0.05 were considered to be statistically significant.

## Results

### Viral infection upregulates TGR5 expression in an IFN/STAT1-dependent manner

**Figure 1:**
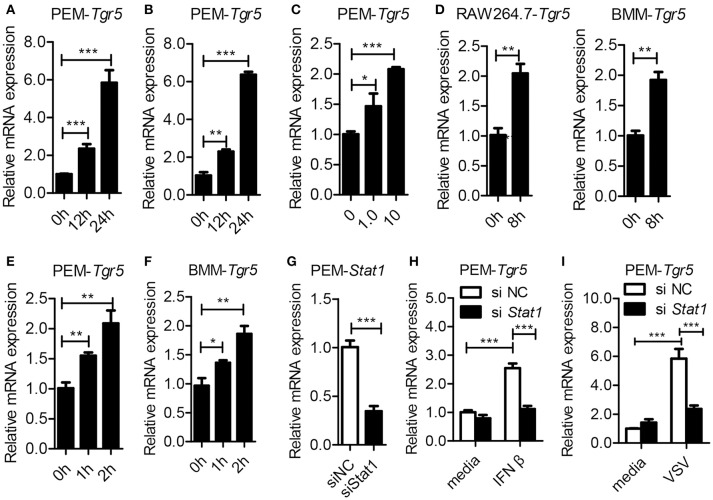
*Tgr5* is an interferon-stimulated gene (ISG). **(A,B)** Quantitative PCR (Q-PCR) analysis of *Tgr5* expression in PEMs infected with RNA virus VSV [1 MOI (multiplicity of infection), **A**] and DNA virus HSV-1 (1 MOI, **B**) for the indicated times. **(C)** Q-PCR analysis of *Tgr5* expression in PEMs infected with the indicated VSV MOI for 8 h. **(D)** Q-PCR analysis of *Tgr5* expression in RAW264.7 cells and BMMs infected with VSV (1 MOI) for 8 h. **(E,F)** Q-PCR analysis of *Tgr5* expression in PEMs **(E)** and BMMs **(F)** stimulated with IFN-β (100 ng/ml) for the indicated hours. **(G)** Q-PCR analysis of *Stat1* expression in PEMs transfected with *Stat1* siRNA for 48 h. NC, negative control. **(H,I)** Q-PCR analysis of *Tgr5* expression in PEMs transfected with *Stat1* siRNA for 48 h and then stimulated with IFN-β (100 ng/ml) for 2 h **(H)** or infected with VSV (1 MOI) for 8 h **(I)**. Glyceraldehyde-3-phosphate dehydrogenase (*GAPDH*) was used as an internal control for Q-PCR. The data are shown as the mean ± SD. *P < 0.05; **P < 0.01; ***P < 0.001. All experiments were performed three times with similar results.

To investigate the potential role of TGR5 in viral infection, we first examined the expression of TGR5 in virus-infected cells. To our surprise, the expression of *Tgr5* increased in mouse peritoneal macrophages (PEMs) infected with vesicular stomatitis virus (VSV) (Figure 1A) or herpes simplex virus type 1 (HSV-1) (Figure 1B). Further, the upregulation of *Tgr5* expression was VSV MOI dependent (Figure 1C). Additionally, VSV infection enhanced *Tgr5* expression in both macrophage-like RAW264.7 cells and bone marrow-derived macrophages (BMMs) (Figure 1D). As IFN-I are robustly induced during viral infection, we next assessed whether TGR5 is upregulated by IFN-I. As shown in Figures 1E,F, the expression of *Tgr5* was enhanced by IFN-β in PEMs and BMMs. To further explore the mechanism of IFN-induced TGR5 expression, we knocked down the expression of *Stat1* in PEMs (Figure 1G) and found that IFN- and VSV-induced *Tgr5* transcription was significantly reduced (Figures 1H,I). Taken together, these data suggest that viral infection upregulates TGR5 expression in an IFN/STAT1-dependent manner, which can be recognized as an interferon (IFN)-stimulated gene (ISG).

### Overexpression of TGR5 inhibits viral replication

**Figure 2:**
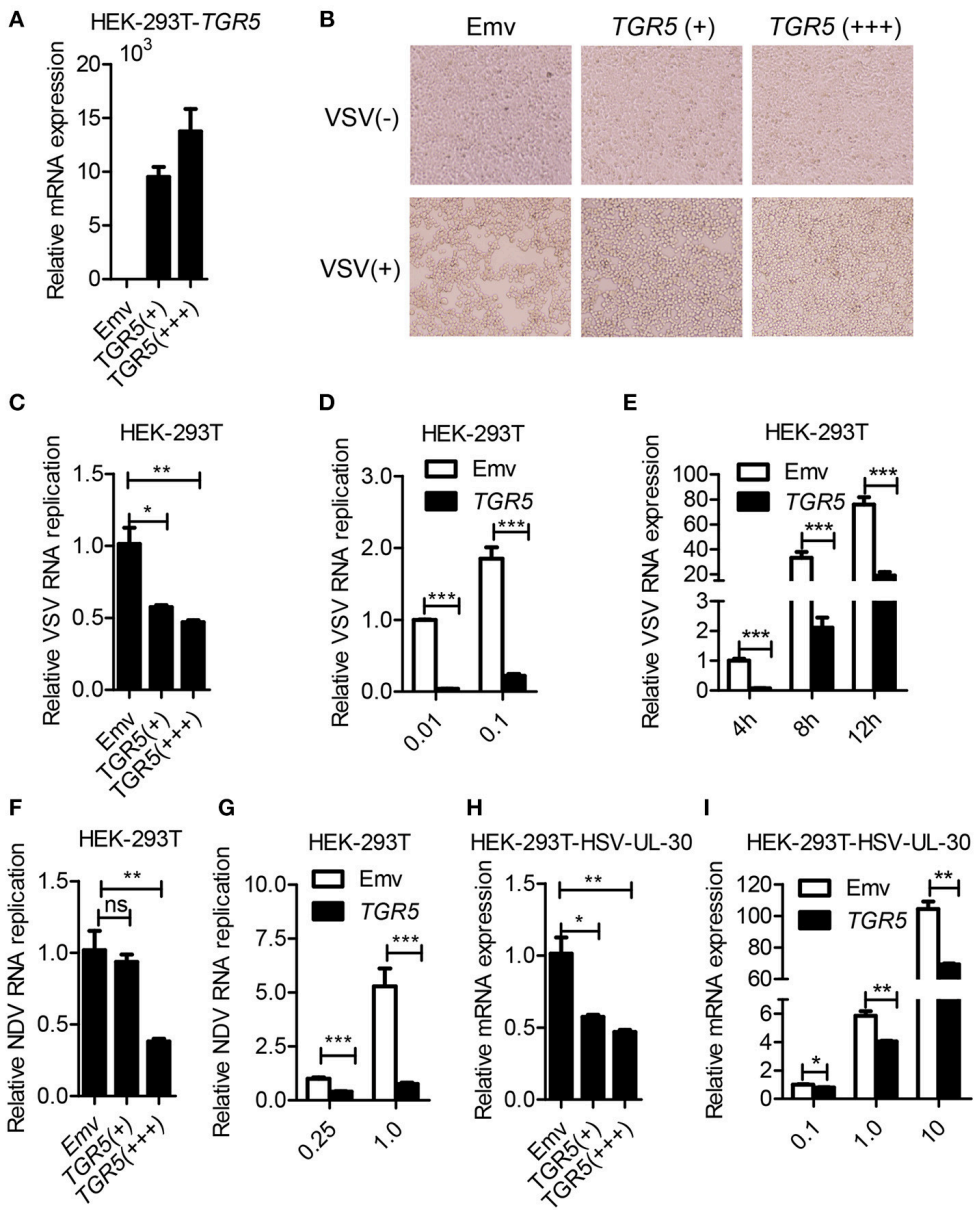
Overexpression of TGR5 inhibits virus replication. **(A)** Q-PCR analysis of *TGR5* expression in HEK-293T cells transfected for 28 h with different amounts (0.6 and 1.8 μg) of TGR5 expression plasmid. Emv, empty vector. **(B)** HEK-293T cells transfected with *TGR5* plasmid as described in **(A)** were infected with VSV (0.1 MOI) for 8 h and then observed under a microscope. Original magnification 10 × . **(C)** Q-PCR analysis of VSV RNA replicates in **(B)**. **(D,E)** Q-PCR analysis of VSV RNA replicates in *TGR5*-overexpressing (1.8 μg) HEK-293T cells infected by VSV with the indicated MOI **(D)** and times **(E)**. **(F)** Q-PCR analysis of Newcastle disease virus (NDV) RNA replicates in HEK-293T cells transfected with *TGR5* plasmids as described in **(A)** and infected with NDV (0.25 MOI) for 12 h. **(G)** Q-PCR analysis of NDV RNA replicates in *TGR5*-overexpressing (1.8 μg) HEK-293T cells infected by NDV with the indicated MOI for 18 h. **(H)** Q-PCR analysis of herpes simplex virus (HSV-1) UL-30 expression in HEK-293T cells transfected with *TGR5* plasmids as described in **(A)** and infected with HSV-1 (0.5 MOI) for 18 h. **(I)** Q-PCR analysis of HSV-1 UL-30 expression in *TGR5*-overexpressing (1.8 μg) HEK-293T cells infected by HSV-1 with the indicated MOI for 18 h. *GAPDH* was used as an internal control for Q-PCR. The data are shown as the mean ± SD. ns, not significant; *P < 0.05; **P < 0.01; ***P < 0.001. All experiments were performed three times with similar results.

To further determine whether TGR5 is a required antiviral factor, we overexpressed TGR5 in HEK-293T cells (Figure 2A) and found that the overexpression of TGR5 increased the viability of HEK-293T cells following VSV infection in a dose-dependent manner (Figure 2B). Accordingly, the replication of VSV was decreased in TGR5-overexpressing HEK-293T cells (Figure 2C). This inhibition of VSV replication by TGR5 was also found at different VSV MOIs (Figure 2D) and times (Figure 2E). To investigate the broad protection of TGR5 in viral infection, we infected TGR5-overexpressing HEK-293T cells with Newcastle disease virus (NDV) and HSV-1. Our data showed that TGR5 significantly reduced the replication of both NDV (Figures 2F,G) and HSV-1 (Figures 2H,I). Thus, as an ISG, TGR5 broadly inhibits the propagation of different viruses.

### TGR5 deficiency promotes viral infection both *in vitro* and *in vivo*

**Figure 3:**
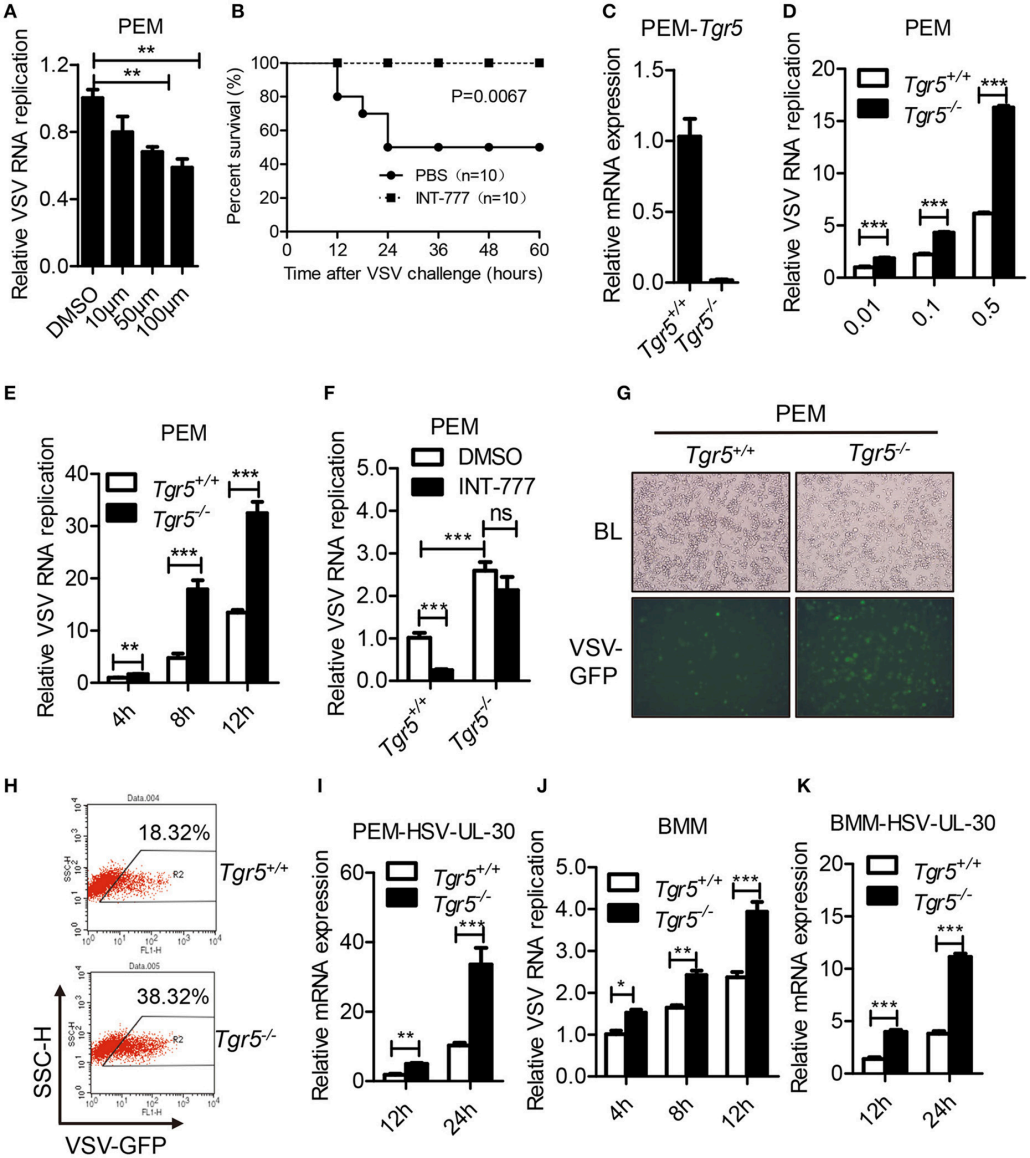
TGR5 deficiency promotes viral infection *in vitro*. **(A)** Q-PCR analysis of VSV RNA replicates in PEMs pretreated with INT-777 for 1 h at the indicated dose (μM) and then infected with VSV (0.1 MOI) for 8 h. DMSO, dimethyl sulfoxide. **(B)** Survival of 8-week-old control or INT-777 (30 mg/kg) pretreated mice given intraperitoneal injections of VSV [1 × 10^8^ plaque-forming units (pfu)/g] (*n* = 10 per group). **(C)** Q-PCR analysis of *Tgr5* expression in *Tgr5*^+/+^- and *Tgr5*^−/−^*-*PEMs. **(D,E)** Q-PCR analysis of VSV RNA levels from *Tgr5*^+/+^- and *Tgr5*^−/−^-PEMs infected with the indicated VSV MOI for 8 h **(D)** and with 1 MOI for the indicated times **(E)**. **(F)** Q-PCR analysis of VSV RNA levels from *Tgr5*^+/+^- and *Tgr5*^−/−^-PEMs pretreated with INT-777 (500 μM) for 1 h and then infected with VSV (1 MOI) for 8 h. **(G,H)** PEMs from *Tgr5*^+/+^- and *Tgr5*^−/−^-mice were infected VSV-GFP (0.01 MOI) for 12 h, and VSV-GFP was examined by light microscopy **(G)** or fluorescence-activated cell sorting (FACS) **(H)**. Original magnification 10 × ; BL, bright light; SSC-H, side scatter-height. **(I)** Q-PCR analysis of HSV-1 UL-30 expression in *Tgr5*^+/+^- and *Tgr5*^−/−^*-*PEMs infected with the HSV-1 (1 MOI) for the indicated times. **(J,K)** Q-PCR analysis of VSV RNA levels **(J)** and HSV-1 UL-30 expression **(K)** in *Tgr5*^+/+^- and *Tgr5*^−/−^*-*BMMs infected with VSV (1 MOI) or HSV-1 (1 MOI) for the indicated times. *GAPDH* was used as an internal control for Q-PCR. The data are shown as the mean ± SD. ns, not significant; *P < 0.05; **P < 0.01; ***P < 0.001. All experiments were performed three times with similar results.

**Figure 4:**
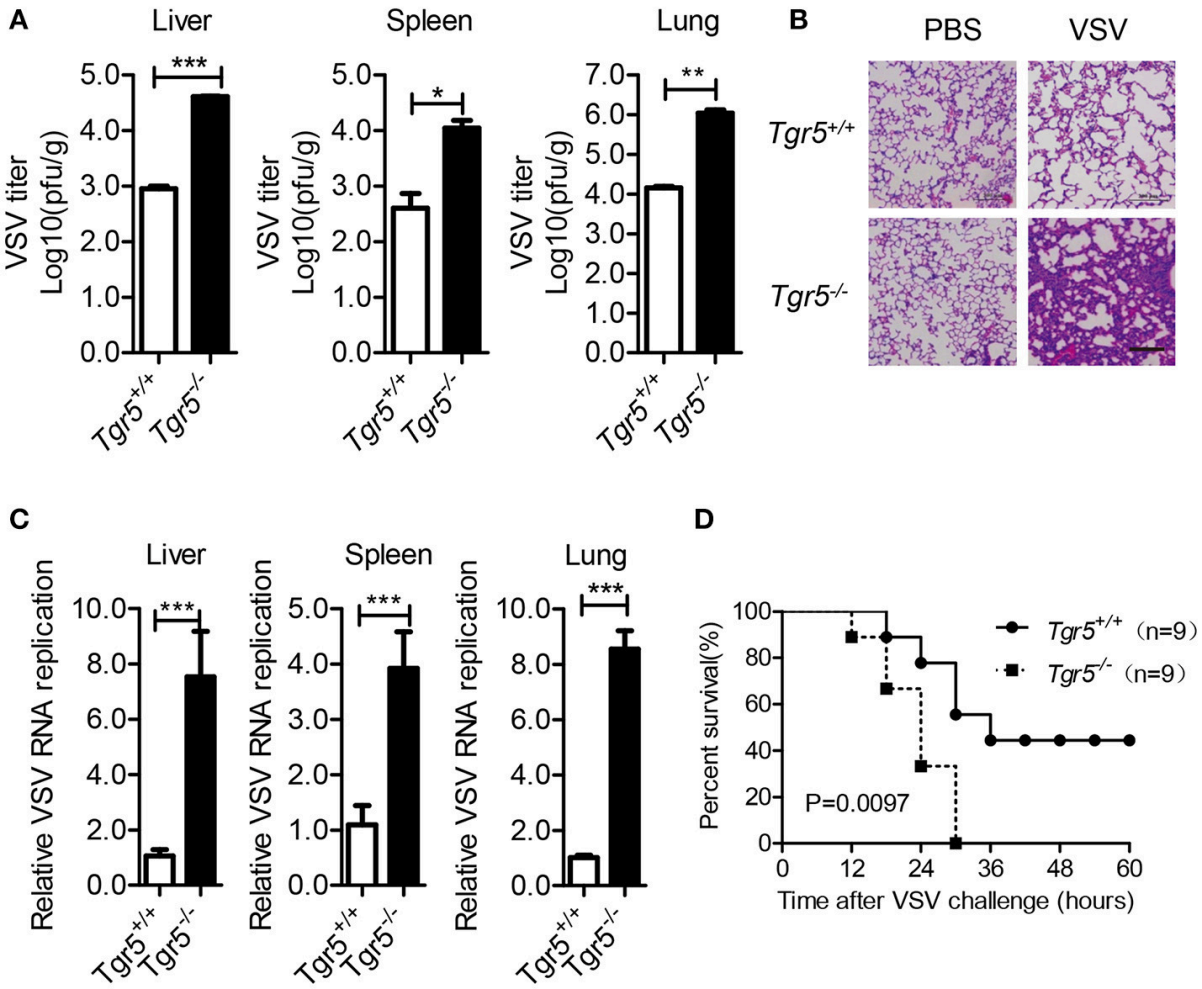
TGR5 deficiency promotes viral infection *in vivo*. **(A)** Determination of VSV loads in organs by standard plaque assays from *Tgr5*^+/+^- and *Tgr5*^−/−^-mice intraperitoneally infected with VSV (1 × 10^8^ pfu per mouse) for 24 h. **(B)** Haematoxylin & eosin staining of lung sections from mice in **(A)**. Scale bar, 200 μm. PBS, phosphate-buffered saline. **(C)** Determination of VSV loads in organs by Q-PCR from *Tgr5*^+/+^- and *Tgr5*^−/−^-mice intravenously infected with VSV (1 × 10^5^ pfu per mouse) for 24 h. **(D)** Survival of 8-week-old *Tgr5*^+/+^- and *Tgr5*^−/−^-mice given intraperitoneal injections of VSV (1 × 10^8^ pfu/g) (*n* = 9 per group). *GAPDH* was used as an internal control for Q-PCR. The data are shown as the mean ± SD. *P < 0.05; **P < 0.01; ***P < 0.001. All experiments were performed three times with similar results.

As a potent agonist, 6α-Ethyl-23(S)-methylcholic Acid (INT-777) was discovered to activate TGR5 intracellular signaling selectively (23). When we pretreated PEMs with INT-777, we found that INT-777 inhibited VSV infection in a concentration-dependent manner (Figure 3A). Most importantly, the survival of INT-777-treated mice was highly increased (Figure 3B). To further confirm the antiviral role of TGR5, we constructed *Tgr5*-knockout mice (Figure 3C). Next, we challenged wild-type and *Tgr5*-deficient PEMs with VSV for the indicated MOIs and times. We observed that TGR5 deficiency significantly increased VSV replication (Figures 3D,E). Meanwhile, we also found that INT-777 inhibited VSV infection in wild-type but not in *Tgr5*-deficient PEMs, which confirmed the specific activation of TGR5 by INT-777 (Figure 3F). In addition to VSV, infection with VSV-GFP and HSV-1 was also increased in *Tgr5*-deficient PEMs (Figures 3G–I). Similar results were also observed in *Tgr5*-deficient BMMs (Figures 3J,K). In order to further determine whether TGR5 mediated viral infection *in vivo*, we intraperitoneally infected *Tgr5*^+/+^- and *Tgr5*^−/−^-mice with high-dose VSV (1 × 10^8^ pfu per mouse) for 24 h. VSV replication in the liver, spleen, and lung were all enhanced (Figure 4A), and the virus-induced lung tissue injury was more serious (Figure 4B) in *Tgr5*-deficient mice compared to controls. Similar results were observed in *Tgr5*^+/+^- and *Tgr5*^−/−^-mice intravenously infected with low-dose VSV (1 × 10^5^ pfu per mouse) (Figure 4C). Accordingly, the post-infection survival of *Tgr5*-deficient mice was much lower than that of their wild-type littermates (Figure 4D). Taken together, these results indicated that TGR5 deficiency promotes viral infection significantly both *in vitro* and *in vivo*.

### TGR5 positively regulates IFN-I production

**Figure 5:**
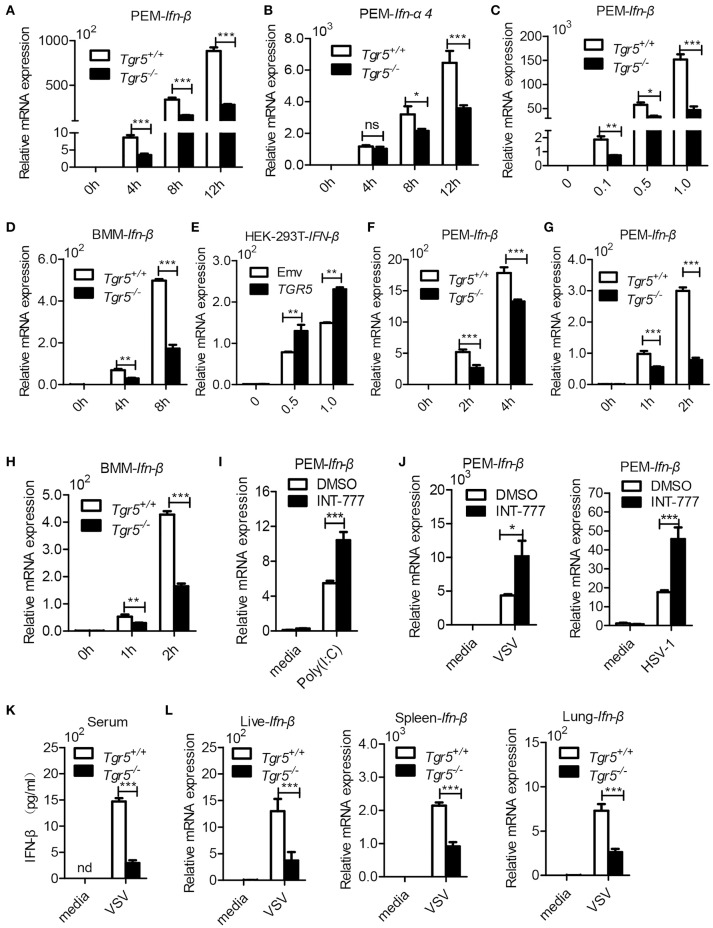
TGR5 positively regulates IFN-I production. **(A,B)** Q-PCR analysis of *Ifn-*β **(A)** and *Ifn-a4* **(B)** expression in *Tgr5*^+/+^- and *Tgr5*^−/−^-PEMs infected with VSV (1 MOI) for the indicated times. **(C)** Q-PCR analysis of *Ifn-*β expression in *Tgr5*^+/+^- and *Tgr5*^−/−^-PEMs infected with the indicated VSV MOI for 8 h. **(D)** Q-PCR analysis of *Ifn-*β expression in *Tgr5*^+/+^- and *Tgr5*^−/−^-BMMs infected with VSV (1 MOI) for the indicated times. **(E)** Q-PCR analysis of *IFN-*β expression in *TGR5*-overexpressing (1.8 μg) HEK-293T cells infected with VSV (1 MOI) for 8 h. **(F,G)** Q-PCR analysis of *Ifn-*β expression in *Tgr5*^+/+^- and *Tgr5*^−/−^-PEMs transfected with Poly (I:C) (1.0 μg/ml) **(F)** or stimulated with Poly (I:C) (10 μg/ml) **(G)** for the indicated times. **(H)** Q-PCR analysis of *Ifn-*β expression in *Tgr5*^+/+^- *and Tgr5*^−/−^-BMMs stimulated with Poly (I:C) (10 μg/ml) for the indicated times. **(I)** Q-PCR analysis of *Ifn-*β expression in PEMs pretreated with INT-777 (500 μM) for 1 h and then stimulated with Poly (I:C) (10 μg/ml) for 2 h. **(J)** Q-PCR analysis of *Ifn-*β expression in PEMs pretreated with INT-777 (500 μM) for 1 h and then infected with VSV (1 MOI) or HSV-1 (1 MOI) for 8 h. **(K)** ELISA of IFN-β in sera from *Tgr5*^+/+^- and *Tgr5*^−/−^-mice intraperitoneally injected with VSV (1 × 10^8^ pfu per mouse) for 24 h. nd, not detected. **(L)** Q-PCR analysis of Ifn-β expression in organs from *Tgr5*^+/+^- and *Tgr5*^−/−^-mice in **(K)**. *GAPDH* was used as an internal control for Q-PCR. The data are shown as the mean ± SD. ns, not significant; *P < 0.05; **P < 0.01; ***P < 0.001. All experiments were performed three times with similar results.

Since IFN-I are important for host defense against viruses, we further examined the expression of IFN-I in *Tgr5*-deficient cells and mice during viral infection. Surprisingly, when we infected wild-type and *Tgr5*-deficient PEMs with VSV, we found that the expression of *Ifn-*β and IFN-inducible *Ifn-*α*4* was significantly reduced in *Tgr5*-deficient PEMs (Figures 5A,B). Accordingly, VSV-induced *Ifn-*β was decreased in a VSV (MOI) dose-dependent manner (Figure 5C). Similarly, VSV-induced *Ifn-*β was also restricted at different time points in *Tgr5*-deficient BMMs (Figure 5D). To further confirm the regulation of TGR5 in IFN-β signaling, we transiently transfected HEK-293T cells with GFP-TGR5 and found that VSV-induced *IFN-*β production was increased in TGR5-overexpressing HEK-293T cells (Figure 5E). Poly (I:C) is widely used to mimic RNA-virus infection and activate the extracellular TLR3 or the intracellular RIG-I downstream signaling pathway (24, 25). Thus, we transfected PEMs with Poly (I:C) to explore the influence of TGR5 on RIG-I-associated signaling and found that the expression of *Ifn-*β was reduced significantly in *Tgr5*-deficient PEMs (Figure 5F). Next, we found that Poly (I:C)-stimulated *Ifn-*β production was inhibited significantly in *Tgr5*-deficient PEMs and BMMs (Figures 5G,H). Accordingly, INT-777 treatment promoted *Ifn-*β expression in Poly (I:C)-, VSV- and HSV-1-induced PEMs (Figures 5I,J). In addition, *Tgr5*-deficient mice produced less IFN-β in serum (Figure 5K) and organs (Figure 5L) than their wild-type littermates in response to VSV infection. Therefore, our data suggest that TGR5 positively regulates IFN-I production which protects host from viral infection.

### TGR5 amplifies IFN-I signaling via AKT-mediated IRF3 activation

**Figure 6:**
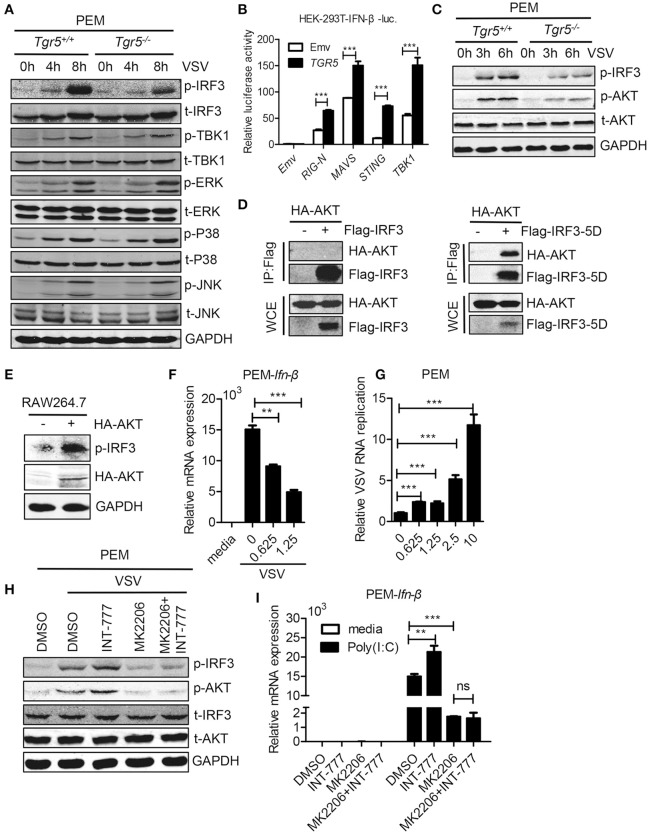
TGR5 amplifies IFN-I signaling via AKT-mediated IRF3 activation. **(A)** Immunoblot analysis of phosphorylated (p-) or total (t-) proteins in lysates of *Tgr5*^+/+^- and *Tgr5*^−/−^-PEMs infected with VSV (1 MOI) for the indicated times. **(B)** TGR5 was co-transfected with RIG-I (N) (RIG-N), MAVS, STING, TBK1, or empty vectors, together with an IFN-β luciferase reporter, into HEK-293T cells for 28 h. IFN-β luciferase activity was detected and normalized to Renilla luciferase activity. **(C)** Immunoblot analysis of phosphorylated (p-) AKT or total (t-) AKT in lysates of *Tgr5*^+/+^- and *Tgr5*^−/−^-PEMs infected with VSV (1 MOI) for the indicated times. **(D)** HEK-293T cells were transfected with plasmids encoding HA-AKT and Flag-IRF3 or Flag-IRF3-5D for 28 h. The cell lysate supernatants were immunoprecipitated using M2 beads, and then immunoblotted with antibodies to HA or Flag tags. WCE, whole-cell extracts. **(E)** Immunoblot analysis of phosphorylated (p-) IRF3 and HA-AKT in lysates of RAW 264.7 cells transfected with plasmids encoding HA-AKT for 28 h. **(F)** Q-PCR analysis of *Ifn-*β expression in PEMs pretreated with MK 2206 (an inhibitor of AKT) at the indicated dose (μM) for 1 h and then infected with VSV (1 MOI) for 8 h. **(G)** Q-PCR analysis of VSV RNA replicates in PEMs pretreated with MK 2206 at the indicated dose (μM) for 1 h and then infected with VSV (1 MOI) for 8 h. **(H)** Immunoblot analysis of phosphorylated (p-) IRF3 and (p-) AKT in lysates of PEMs pretreated with MK 2206 (3 μM) or INT-777 (500 μM) for 1 h, and then infected with VSV (1 MOI) for 8 h. **(I)** Q-PCR analysis of *Ifn-*β expression in PEMs pretreated with MK 2206 (3 μM) or INT-777 (500 μM) for 1 h, and then stimulated with poly (I:C) (10 μg/ml) for 2 h. *GAPDH* was used as an internal control for Q-PCR. The data are shown as the mean ± SD. ns, not significant; **P < 0.01; ***P < 0.001. All experiments were performed three times with similar results.

To investigate the potential mechanism of TGR5 in regulation of IFN-I production, we examined changes in several different signaling pathways during viral infection. We found that only IRF3 phosphorylation was reduced significantly while the phosphorylation of TBK1, ERK, P38, and JNK were all unchanged in *Tgr5*-deficient PEMs (Figure 6A), suggesting a dominant role of IRF3 in TGR5-mediated immune regulation. Accordingly, TGR5 significantly promoted RIG-I (N)-, MAVS-, STING- and TBK1-activated IFN-β-luciferase activity (Figure 6B). Previous studies have shown that TGR5 regulates monocyte adhesion and macrophage migration through the activation of AKT signaling (26, 27), so we next investigated whether AKT is involved in TGR5-regulated IFN-I production. Surprisingly, we found that the phosphorylation of AKT was reduced in *Tgr5*-deficient PEMs following VSV infection (Figure 6C). Interestingly, several papers have demonstrated that AKT is involved in IRF3 activation and AKT only binds to IRF3-5D (a constitutively active form of IRF3) (28, 29). Using co-immunoprecipitation, we found similar results (Figure 6D), and that the phosphorylation of IRF3 could be increased markedly after overexpressing HA-AKT in RAW264.7 macrophages (Figure 6E). In addition, we also observed that VSV-induced *Ifn-*β expression was reduced (Figure 6F) and VSV replication was increased (Figure 6G) by pretreating the PEMs with the AKT inhibitor MK2206. Most importantly, the induction of phosphorylated IRF3 by INT-777 in VSV-infected PEMs was blocked by MK2206 (Figure 6H). Likewise, the promotion of *Ifn-*β expression by INT-777 in Poly (I:C)-stimulated PEMs was also abolished by MK2206 (Figure 6I). Taken together, these results indicated that TGR5 enhances IFN-I signaling via AKT-mediated IRF3 activation.

## Discussion

In this study, we describe a positive feedback mechanism for the regulation of antiviral innate immune responses by metabolite-sensing TGR5. Viral infection upregulates TGR5 expression in an IFN/STAT1-dependent manner which in turn amplifies the antiviral innate immune responses via AKT-mediated IRF3 activation. Interestingly, previous studies have shown that bile acid biosynthesis was significantly increased in HCV and HBV infection suggested that bile acids may be positive signals in fighting viral infection (30, 31). However, TGR5 also negatively regulates NF-κB signaling (32) and NLRP3 inflammasome activation (33). The difference between inflammatory responses and antiviral innate immune responses suggests that TGR5 plays complex roles in immune responses. It is well known that respirovirus infection-induced acute lung injury is the main reason for clinical mortality. So finding a novel therapeutic target to restrict the viral replication and virus induced inflammation at the same time would be quite valuable.

IFN-I are rapidly activated during viral infection and broadly inhibit growth of many types of virus (34). As a pivotal mediator of host defense against viral challenges, IFN-I establish a cellular antiviral state mainly through upregulation of hundreds of ISGs, which could interfere with different viruses through distinct mechanisms (35). Almost 2,000 human and mouse ISGs have been identified from microarray datasets, most of which remain uncharacterized in viral infection (36). Hence, further elucidation of the functions and mechanisms of these ISGs will contribute to new and more efficacious therapeutics for viral infection. Here, we demonstrated that the expression of TGR5 is increased by viral infection or IFN-β, and that a broad range of viruses was restricted by TGR5 through increased IFN-I production which constitutes a positive feedback response for antiviral immunity. Furthermore, increased bile acid biosynthesis following viral infection could also facilitate this positive feedback through specifically activating TGR5 signaling. This may be an evolutionary strategy for hosts to achieve a global antiviral effect.

As a membrane receptor, binding of ligands such as bile acids to TGR5 results in the activation of the adenylyl cyclase signaling pathway and other cell-specific activation signaling cascades (37). In our work, we found that TGR5 promotes IFN-I production via AKT-mediated IRF3 activation during viral infection in macrophages. And we also observed that AKT only binds to IRF3-5D which is a constitutively active form of IRF3, which suggests that AKT-mediated positive regulation of IRF3 is phosphorylation dependent. So it's possible that the IRF3 signaling activated upon viral infection increases the expression of IFN-β. Then the IFN-β enhances the expression of TGR5 and bile acid biosynthesis to activate AKT signaling through TGR5 to synergistically amplify the IRF3 signaling, constituting a positive feedback loop for IFN-β signaling. Thus, our findings reveal an IFN-β and metabolite-sensing receptor-mediated positive feedback loop in antiviral immune responses.

The metabolic control of immune responses is critical for the maintenance of immune homeostasis and immune disorders. Pathogenic infections induce metabolic reprogramming through different pathways to meet the energy and metabolite demands for pathogen propagation. Thus, a breakdown of metabolic balance in viral infection should be precisely sensed by the host. Here we demonstrated that TGR5 deficiency enhances virus infection, which is similar to pharmaceutical inhibition of FXR which results in increased viral replication (38), implying that bile acids could be a novel host antiviral metabolite. Interestingly, our data also showed that TGR5 could be increased by interferon and further demonstrated the great potential of bile acids and its receptor in fighting against virus, emphasizing the specific role of interferon in rewiring cellular metabolism to activate immune cells and limit viral infection.

## Author contributions

BD, MQ, and ML supervised the project. BD, HHu, and QX conceived and designed the experiments. QX, HHu, NW, RC, and NC performed the experiments. BD, HHu, QX, QW, and HHa analyzed the data. BD, HHu, QX, and SS wrote the manuscript.

### Conflict of interest statement

The authors declare that the research was conducted in the absence of any commercial or financial relationships that could be construed as a potential conflict of interest.
